# Impact of interventions on malaria in internally displaced persons along the China–Myanmar border: 2011–2014

**DOI:** 10.1186/s12936-016-1512-2

**Published:** 2016-09-15

**Authors:** Guofa Zhou, Eugenia Lo, Daibin Zhong, Xiaoming Wang, Ying Wang, Sameer Malla, Ming-chieh Lee, Zhaoqing Yang, Liwang Cui, Guiyun Yan

**Affiliations:** 1 University of California, Irvine, CA USA; 2 Southern Medical University, Guangzhou, China; 3 Kunming Medical University, Kunming, China; 4 Pennsylvania State University, University Park, PA USA; 5 Third Military Medical University, Chongqing, China

**Keywords:** Malaria, Outbreak, Internally displaced persons, Intervention, China–Myanmar border

## Abstract

**Background:**

Internally displaced persons (IDP) represent vulnerable populations whose public health conditions merit special attention. In the China–Myanmar border area, human movement and resettlements of IDP can influence malaria transmission. Comparison of disease incidence and vector densities between IDP camps and surrounding local villages allows for better understanding of current epidemiology and to evaluate the effectiveness of interventions in the region.

**Methods:**

Malaria and vector surveillance was conducted in three IDP camps and three local villages neighbouring the camps along the China–Myanmar border in Myanmar. Clinical malaria cases were collected from seven hospitals/clinics from April 2011 to December 2014. Malaria vector population dynamics were monitored using CDC light traps. The use of malaria preventive measures and information on aid agencies and their activities was obtained through questionnaire surveys.

**Results:**

Malaria was confirmed in 1832 patients. Of these cases, 85.4 % were *Plasmodium vivax* and 11.4 % were *Plasmodium falciparum* malaria. Annual malaria incidence rates were 38.8 and 127.0 cases/1000 person year in IDP camps and local villages, respectively. Older children of 5–14 years had the highest incidence rate in the camps regardless of gender, while male adults had significantly higher incidence rates than females in local villages and females child-bearing age had significantly lower risk to malaria in IDP camps compare to local villages. Seasonal malaria outbreaks were observed both in the IDP camps and in the local villages from May to August 2013. The proportion of *P. vivax* remained unchanged in local villages but increased by approximately tenfold in IDP camps from 2011 to 2014. *Anopheles* vector density was tenfold higher in local villages compared to IDP camps (2.0:0.2 females/trap/night). Over 99 % of households in both communities owned bed nets. While long-lasting insecticidal nets accounted for 61 % of nets used in IDPs, nearly all residents of local villages owned regular nets without insecticide-impregnation. There were more active aid agencies in the camps than in local villages.

**Conclusion:**

Malaria in IDP camps was significantly lower than the surrounding villages through effective control management. The observation of *P. vivax* outbreaks in the study area highlights the need for increased control efforts. Expansion of malaria intervention strategies in IDP camps to local surrounding villages is critical to malaria control in the border area.

**Electronic supplementary material:**

The online version of this article (doi:10.1186/s12936-016-1512-2) contains supplementary material, which is available to authorized users.

## Background

Global efforts to eliminate and eradicate malaria have highlighted the need to target control efforts in international border regions endemic for to malaria [1]. In addition to cross-border migrants seeking economic opportunities, settlements for refugees and internally displaced person (IDP) along international borders as a result of internal conflicts can influence malaria transmission [2]. Refugees and IDPs generally settle in over-crowded suboptimal living conditions, placing them at an increased risk of infectious diseases, particularly water-borne enteric disease and vector-borne disease [3–9]. Disease morbidity in camps and settlements can be greatly reduced if appropriate preventive and treatment measures are implemented in a timely manner [10–17].

Malaria remains one of the most concerning infectious diseases among displaced populations [4, 5, 18–22]. In Southeast Asia, malaria is a significant problem in Myanmar as well as its neighbouring countries through border migrants and refugees [14, 23–26]. Myanmar has the highest malaria burden among other Southeast Asian countries with approximately 200,000 cases per year [26, 27]. Large-scale human movement has led to intensive transmission of malaria in the IDP settlement along the Myanmar–Thailand border [28–30]. Along the China–Myanmar border, despite high malaria incidence in the surrounding villages in Myanmar, malaria in IDPs as well as the impact of human movement on malaria transmission are unclear [25, 31].

Following the conflict between the Kachin Independence Army (KIA) of the Kachin State and the Myanmar government armed forces in 2011, hundreds of thousands of civilians have fled their homes and moved to the IDP camps along the China–Myanmar border. These IDP camps, established in July 2011, were administered by the United Nations High Commissioner for Refugees (UNHCR) with assistance from several non-governmental organizations (NGOs). While there have been extensive studies on malaria epidemiology and vector ecology in the local area [25, 31–40], the malaria situation and its long-term impact on public health in the IDP camps along the China–Myanmar border remains largely unknown.

To assess the burden of malaria in the China–Myanmar border area, clinical malaria was monitored prospectively, clinical malaria incidence rates were compared between IDP camps and local villages surrounding the IDP camps. Malaria vector population was monitored, and malaria control and prevention measures in both the IDP camps and local villages were investigated with the goal to explain differences in malaria incidence rates.

## Methods

### Study population

**Fig. 1:**
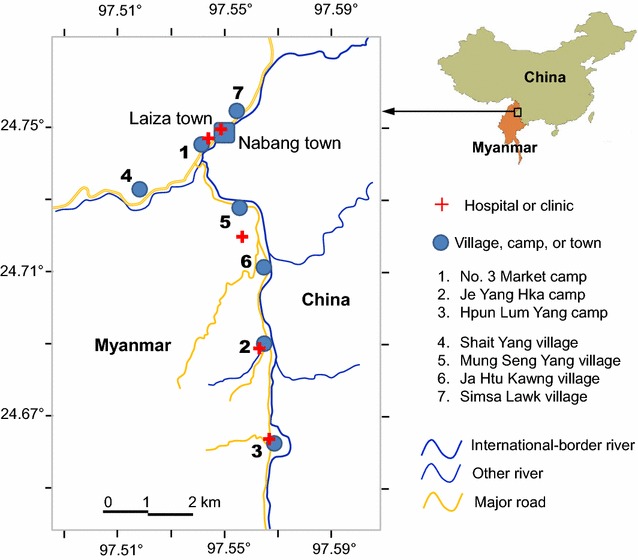
Map of study area. All villages in the area have been mapped as gray patches. Locations of study villages and camps were marked by numbers and clinics/hospitals were marked by red cross. The hospital between sites 5 and 6 is primarily serving for the military

**Fig. 2:**
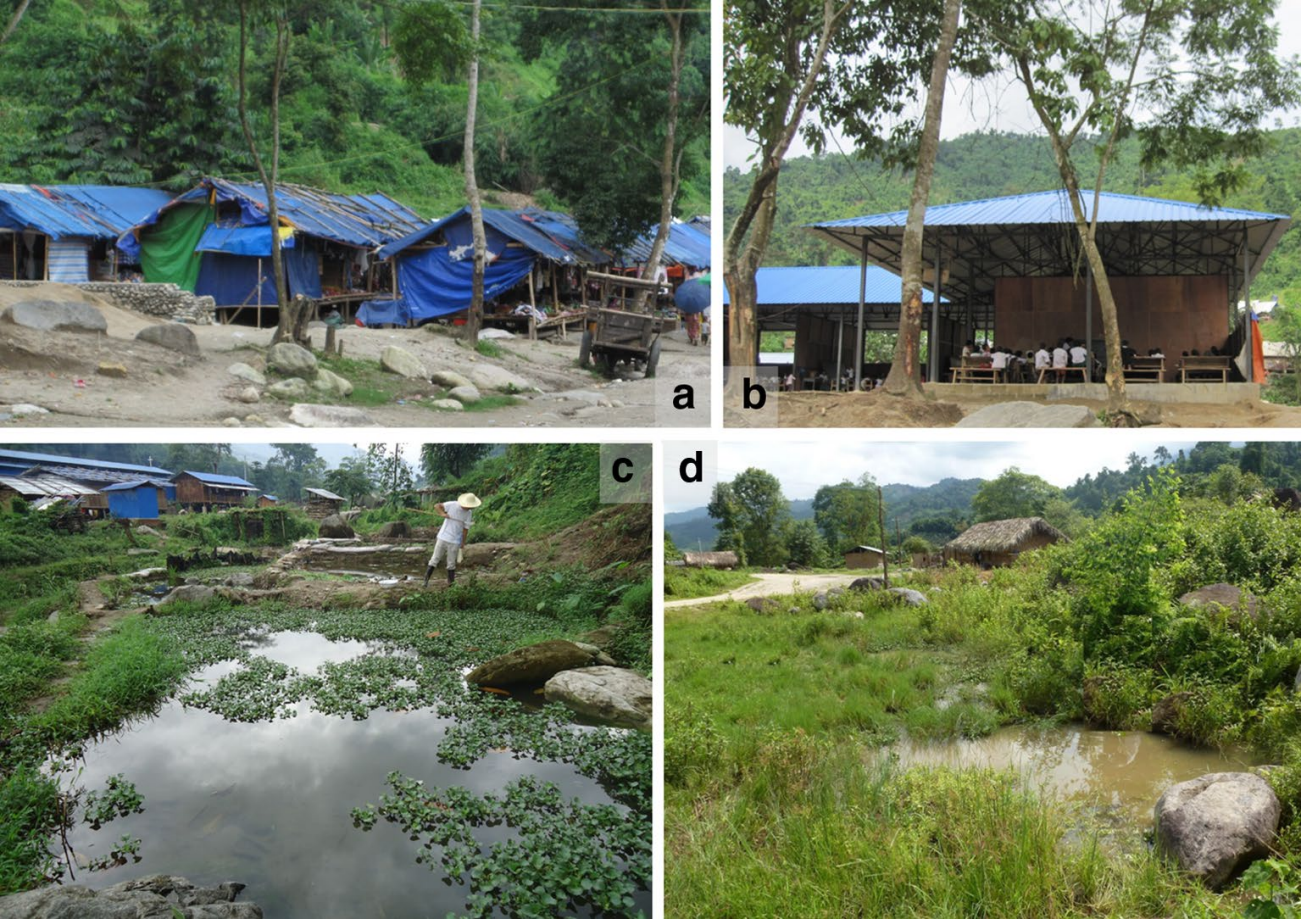
**a** A corner of Je Yang camp; **b** School in Je Yang camp; **c** Typical breeding habitat in Je Yang camp; and **d** Typical thatch-roofed house and mosquito breeding habitat in Je Htu Kawng village

The study was initiated in April 2011 in the China–Myanmar border area of the Kachin state, Myanmar, as part of the International Centers of Excellence for Malaria Research (ICEMR) Southeast Asia project [25, 31]. Shortly thereafter, civil war prompted fleeing populations to resettle in camps by the border areas. In July–August 2011, about 1 month after the camps were established, study areas were expanded to include three IDP camps, located approximately 1.5–4 km away (Figs. 1, 2). By August 2012, catchment population sizes were approximately 11,000 and 1200 in the three IDP camps and four local villages, respectively (Additional file 1). The seven sites are the only residential areas (except Laiza town) on the Myanmar side along the Myanmar–China border, they are not randomly selected sites (Fig. 1), and clinical malaria incidence rate is very low on the China side of the border area [25].

All study sites are located in the same valley area with an elevation between 240 and 280 m above sea level, they have similar landscape and ecological settings [39]. All IDP camps have clinics located within them that provide routine diagnosis and treatment to address the population’s health needs free of charge. The neighbouring study villages have no health clinics and villagers need to travel to nearby health care facilities to seek treatment, usually in Laiza town or less commonly the small military clinic (between Mung Seng Yang village and Ja Htu Kawng village on Fig. 1) before the establishment of IDP camps (Fig. 1). Clinics in IDP camps also provide free diagnosis and treatment for local villagers should they come.

### Clinical malaria surveillance

Passive case surveillance (PCS) was conducted prospectively at all IDP camp clinics and at all hospitals in the Laiza area where residents from the local villages sought treatment. All patients are traced back to the village/camp where they are originally from based on clinical case report and questionnaire surveys. Final data analysis was restricted to consenting PCS cases residing in the study camps and villages (note: in this study, all patients with malaria symptoms were asked to sign the consent form before been recruited, all of them have actually signed the consent form). After obtained consents or ascents (for minors <18 years) from the patients or parents/guardians, finger-prick blood samples were obtained from individuals who had malaria-like symptoms, and thick and thin smears were prepared to identify malaria parasites, parasite density and species. Clinical malaria was defined as fever (axillary temperature ≥37.5 °C), chills, severe malaise, and headache or vomiting at the time of examination, or 1–2 days prior to the examination, with a *Plasmodium* positive blood smear [25, 41]. Malaria parasites were identified microscopically by two experienced technicians. For quality control purposes, approximately 5 % of the slides were randomly selected for parasite identification by a third microscopist. Individuals found positive for malaria infection and having malaria symptoms were treated by local doctors the same day when possible, if not, the following day according to the World Health Organization guidelines, i.e., chloroquine for the treatment of vivax malaria, dihydroartemisinin–piperaquine for the treatment of uncomplicated falciparum malaria and quinine for falciparum with complications [36, 37]. Primaquine 8-day treatment (0.375 mg/kg/day) was given as radical treatment for vivax [37], however, primaquine single dose was not given as a gametocytocidal for falciparum malaria [36]. Both local villages and IDP camps used the same treatment regimens. However, primaquine treatment of vivax was supervised for patients from IDP camps but not for local villagers due to the difficulties for follow-ups.

Case report forms were administrated to collect the following information from patients: demographic characteristics, occupation, education level, clinical symptoms, history of malaria in the preceding 12 months, history of travel within the 2 weeks preceding the clinic visit, history of fever (days with fever before seeking treatment), and use of measures to prevent malaria. Patient reported revisits of the same case or clinical case follow-up was recorded as one case, however, relapse or recrudescent cases were not confirmed in laboratory.

### Malaria vector population sampling

Longitudinal adult mosquito surveys were initiated in April 2012 in two villages and one IDP camp. Twice a month, at least 36 houses were systematically selected at each site (camp or village) to maximize the coverage for alternate monthly adult mosquito sampling. On average, 147 trap-nights were conducted in each site each month. Mosquitoes were collected using unbaited CDC light traps. *Anopheles* species were morphologically identified. Mosquitoes were pooled monthly and mosquito density was calculated as *Anopheles* females per trap per night (f/t/n).

All potential habitats were identified through thoroughly search over the study area. Mosquito larvae were surveyed using a standard dipper (size of 350 mL). Water was dipped up to 20 times. When a habitat was too small to make 20 dips, water was dipped as many times as possible. Tire tracks, hoof prints and container habitats were not sampled. A subset of larvae was further analysed with the ribosomal DNA (rDNA) polymerase chain reaction (PCR) method [39]. Larvae density was calculated as *Anopheles* larvae per dip per day (l/d/day). Larval sampling was conducted monthly during major malaria transmission seasons, i.e., from April to August, 2012 at all sites and from May to August, 2014 in IDP camps only.

### Malaria preventive questionnaire survey

At each site, we conducted a baseline census in August 2011, followed by 2–3 subsequent surveys per year to update demographic data. In 2013, after obtaining written informed consent, 100 households (519 individuals) from the villages and 300 households (1629 individuals) from the camps were randomly selected and interviewed to gather information on the usage of malaria preventive measures, including bed net ownership, type, number, and usage, indoor residual spray, and travel history during the previous 2 weeks. The sample size was chosen to maximize the representatives of the study populations.

Aid agencies play an important role in IDP camps, providing various supplies and services, as well as disease prevention measures such as indoor residual spraying (IRS) and insecticide-treated bed nets (ITNs). NGO and local government supported projects/services in IDP camps and local villages were investigated in November 2013 through questionnaire surveys. Services were tracked back to 2011. At each site, we interviewed five people, including village/agency staff/head/manager or school head/teacher, and asked them to complete the questionnaire to the best of their knowledge.

### Statistical analysis

Malaria incidence rate was calculated as the number of clinical episodes per 1000 person-years (or months). Population size used to determine incidence rate was based on the 11 demographic surveys, and assumed a constant rate of change in population size between surveys. Statistical significance of differences in monthly malaria incidence rates and vector densities between the IDP camps and the local villages were assessed using a one-way ANOVA post hoc Tukey HSD test with repeated measures. Age- and sex-specific incidence rates were compared between IDP camps and local villages using χ^2^ test and odds ratio was calculated.

### Scientific and ethical statement

Scientific and ethical clearance was obtained from the ethical review boards of Kunming Medical University, China (IRB # KMC2011-01); University of California, Irvine (IRB HS # 2012-9123) and Pennsylvania State University (IRB # 34319), USA. Written informed consent/assent (for minors under age 18) for study participation was obtained from all consenting heads of households and each individual who was willing to participate in the study.

## Results

### Malaria incidence rate

During the survey period, there were 1462 confirmed malaria cases in the IDP camps and 441 cases in the local villages. The annual clinical malaria incidence rate in the villages was on average 127 cases per 1000 person-years, which was significantly higher than that in the camps (38.8 cases per 1000 person-years, adjusted relative risk ratio 3.9, 95 % CI [1.9, 15.9], *P* < 0.0001) (Table 1). In the local villages, 75.1 % of the confirmed cases were *P. vivax*, 22.9 % *P. falciparum*, and 1.8 % mixed infections of *P*. *falciparum* and *P*. *vivax*, while in the IDP camps, 90.4 % were *P. vivax*, 7.9 % *P. falciparum*, and 1.4 % mixed infections. In contrast to villages, the IDP camps exhibited a fourfold higher *P. vivax* infection incidence than *P. falciparum* (odds ratio *OR* = 3.9, 95 % CI [2.9, 5.2], *P* < 0.0001) (Table 2). *Plasmodium malariae* (n = 5) and *Plasmodium ovale* (n = 1) infections were uncommon.

**Table 1:** Malaria incidence rate by gender, age and surveillance time in different study areas

Parameter	Category	IDP camp		Local village		Risk ratio (95 % CI) village/camp
		Incidence rate^a^	Odds ratio (95 % CI)	Incidence rate^a^	Odds ratio (95 % CI)	
Overall		38.80		127.0		3.32 [2.91, 3.61]***
Gender	Male	44.15	1	126.45	1	2.86 [2.59, 3.17]***
	Female	37.48	0.82 [0.73, 0.92]***	60.71	0.31 [0.24, 0.40]***	1.62 [1.38, 1.91]***
Age (years)	0–4	34.38	1	88.00	1	2.56 [1.95, 3.36]***
	5–14	69.56	2.29 [1.92, 2.73]***	78.72	0.85 [0.55, 1.30]	1.13 [0.96, 1.34]
	≥15	26.06	0.69 [0.58, 0.83]***	103.96	1.31 [0.88, 1.95]	3.99 [3.53, 4.50]***
Female age (years)	15–45	28.19	1	65.52	1	2.33 [1.82, 2.97]***
	Other	40.72	1.53 [1.28, 1.83]***	53.79	0.77 [0.52, 1.14]	1.32 [1.06, 1.65]*
Year	2011	14.48	1	29.33	1	2.03 [1.38, 2.98]***
	2012	15.98	1.12 [0.87, 1.43]	74.81	2.68 [1.77, 4.05]***	4.68 [3.61, 6.07]***
	2013	88.02	6.64 [5.46, 8.08]***	141.65	5.46 [3.72, 8.02]***	1.61 [1.39, 1.87]***
	2014	30.81	2.19 [1.77, 2.71]***	118.00	4.43 [2.98, 6.57]***	3.83 [3.17, 4.63]***

*, *** Significant different at level of 5 and 0.1 %, respectively

^a^Incidence rate is defined as malaria cases per 1000 people year

**Table 2:** Temporal changes in *P. vivax* over *P. falciparum* ratio (Pv/Pf ratio) in IDP camps and local villages

Year	IDP camp		Local village		Camp vs. village
	Pv/Pf	Odds ratio (95 % CI)	Pv/Pf	Odds ratio (95 % CI)	Rate ratio (95 % CI)
2011	2.09	1	1.50	1	1.13 [0.82, 1.56]
2012	3.22	1.54 [0.87, 2.73]	2.95	1.97 [0.81, 4.76]	1.09 [0.58, 2.06]
2013	21.87	10.46 [6.17, 17.72]***	3.16	2.11 [0.94, 4.71]	6.92 [4.33, 11.07]***
2014	24.46	11.70 [5.85, 23.38]***	4.67	3.11 [1.33, 7.30]**	5.24 [2.58, 10.65]***

**, *** Significant different at level of 1 and 0.1 %, respectively

**Fig. 3:**
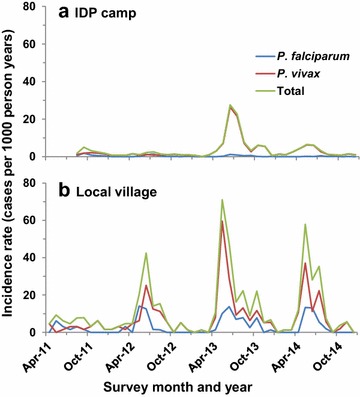
Monthly dynamics of clinical malaria incidence rates (cases per 1000 person years) in IDP camps (**a**) and local villages (**b**) from 2012 to 2014. Total represent incidence rate of *P. falciparum* alone, *P. vivax* alone, and sum of all parasite species, respectively

The monthly dynamics of malaria incidence rates varied significantly between the IDP camps and local villages over time (Fig. 3). Clinical malaria incidence rates in the camps were low in the first 21 months after the camps were established, with an average monthly incidence rate of 1.7 cases per 1000 person-years (*P. falciparum* 21.2 % and *P. vivax* 71.6 %) (Table 2). Between May and August 2013, the IDP camps experienced an epidemic with an overall monthly incidence rate of 16.3 cases per 1000 person-years (*P. falciparum* 4.5 % and *P. vivax* 95.3 %) (Table 2), tenfold higher compared to previous years (Fig. 3), and the increase in *P. vivax* was more pronounced than that in *P. falciparum* (Table 2). Subsequently, it dropped to an average monthly incidence of 2.9 cases per 1000 person-years from September 2013 to the end of 2014. By contrast, the local villages consistently exhibited high malaria incidence rates during the months of May–August every year, and the peak incidence rates varied from year to year with the highest in 2013 (Fig. 3). A large proportion of the malaria cases in the IDP camps was due to *P. vivax,* and the ratio of *P*. *vivax* and *P*. *falciparum* cases increased over time, from 2.1 in 2011 to 24.5 in 2014. On the contrary, the ratio of *P*. *vivax* and *P*. *falciparum* cases remained constant in the villages over the four years (Table 2).

Males had significantly higher malaria incidence rates than females in the local villages, but not in the IDP camps (Table 1). In IDP camps, older children (5–14 years) had the highest malaria incidence rate among all age groups; while adults (≥15 years) had the highest incidence rate in local villages (Table 1). Overall, women of child-bearing age (15–45 years) were at considerably higher risk of malaria compared to other females. There was slight difference in age- and gender-specific incidence rates between *P. falciparum* and *P. vivax* (Additional file 2). For example, for *P. falciparum* malaria, male adults were the most vulnerable group in both IDP camps and local villages, while gender was not a risk factor for vivax malaria in IDP camps (Additional file 2). Prompt diagnostic and treatment of *P. falciparum* clinical cases is the main factor that influences transmission. In local villages, the mean days with malaria symptoms before seeking treatment for uncomplicated falciparum malaria was 3.22 d (95 % CI 3.22 ± 0.63, median 3, range 1–10 days), which is not significantly different from that in IDP camps (mean 3.00 ± 0.25, median 3, range 1–7 days) (two-samples t test assuming unequal variances t = 0.66, df = 46, P = 0.26).

### Malaria vector population density

**Fig. 4:**
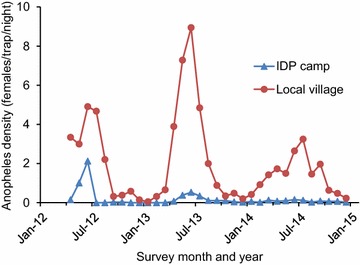
Monthly dynamics of *Anopheles* densities (*Anopheles* females/trap/night) in IDP camps and local villages from 2012 to 2014

The predominant vector species was *Anopheles minimus* (85 % in local villages and 81 % in IDP camps). Other major vector species included *Anopheles maculatus* s.l. (3.4 %), *Anopheles culicifacies* s.l. (2.8 %), *Anopheles jeyporiensis* (1.8), *Anopheles vagus* (1.4 %) and *Anopheles sinensis* (1.0 %), and eight other malaria vector species with <1 % to total vector population. On average, *Anopheles* adult density was tenfold higher in local villages than in the IDP camps (2.00:0.18 f/t/n, Tukey HSD test *P* < 0.001). The vector density in both IDP camps and local villages decreased from 2012 to 2014 (Fig. 4). In IDP camps, vector density decreased about fivefold from 0.37 f/t/n in 2012 to 0.14 f/t/n in 2013 and 0.08 f/t/n in 2014. However, the decrease in vector densities was not statistically significant in both settings (ANOVA with repeated measure, for IDP camps, *F*_2,30_ = 1.51, *P* = 0.23; for local villages, *F*_2,30_ = 0.82, *P* = 0.45).

Larval survey indicated that there was no significant difference in average number of breeding habitats (18.0:18.5), habitat infestation rate (34.7%:36.1 %) and mean larval density (0.19:0.32 larvae/dip) between IDP camps and local villages in 2012. However, different from the decreasing trend in adult density, larval density in the IDP camps was significantly higher in 2014 than that in 2012 (larval density 0.70:0.19 larvae per dip, *t* = 3.44, df = 11, P < 0.01). In addition, more breeding habitats have been found in IDP camps in 2014, monthly average of 26.3 habitats, than that in 2012.

### Disease preventive methods used

Among the 400 households surveyed (100 in local villages and 300 in IDP camps), 100 % used at least one malaria prevention method (including ITNs, IRS, and repellents), and only one household (0.3 %) in the IDP camps did not own an ITN. Bed net usage, defined as the percentage of individuals who slept under nets, was 76.4 % in IDP camps and 75.9 % in local villages (*P* = 0.81). However, the IDP camps and local villages used different types of bed nets. In the IDP camps, 60.9 % of bed nets were long-lasting insecticide-treated nets (LLIN) whereas 0.4 % used LLIN in local villages.

More than ten NGOs and local agencies have provided various services in the IDP camps, while fewer aid agencies have provided limited services in the local villages (Additional file 3). Some local governmental agencies and NGOs resided in the camps, but all aid agencies in local villages only stayed temporally, usually 1–3 months. In all study villages and camps, free ITNs and insecticide spray were provided, but insecticide spray was more frequent and reliable in camps than in local villages (Additional file 4). In local villages, insecticide spray was done once a year in summer, whereas in IDP camps, indoor spray was routine almost monthly and ground and outdoor spray was done four times a year. Spray team in IDP camps was ready to treat anywhere at any time when needed. Disease diagnosis and treatment were also free for everyone in all clinics and hospitals, i.e., local villagers can also visit hospitals in the camps free of charge. While clinics and doctors/nurses were located in the camps, there was no clinic and doctor/nurse in the local villages (Additional file 4). Local villagers have to go to either hospitals in the camps or elsewhere far away from home.

## Discussion

The observations from this study indicate that the recently established IDP camps had a significantly lower burden of malaria compared to local villages in the same area. Prompt establishment of health care clinics, resource mobilization by international and non-governmental agencies in response to the disaster, and pro-active malaria control activities such as indoor and outdoor residual spray and adequate case management could all be factors affecting the risk of malaria transmission in the camps. Early diagnosis and treatment potentially played more vital role in malaria control than ITNs in such context like this study, where the major vector *An. minimus* can feed on both humans and animals, and inhabit/feed both indoors and outdoors [42, 43]. For patients with falciparum infections, there was no difference in duration of fever days before seeking treatment between local villagers and IDPs. No primaquine was used in both areas for falciparum malaria treatment, and the current treatment regimens are rather effective [36]. The decreased trend and lower *P. falciparum* incidence rate in IDP camps (but not the case in local villages) may be explained by less frequent human movement in the camp area compared to residents in the villages. Moreover, effective vector interventions may also reduce the overall *P. falciparum* transmission in the camps. Although primaquine has been administrated as radical treatment for vivax in both local villages and IDP camps, only patients from IDP camps were closely supervised for such treatment through follow-up visits. This practice may have major effects in alleviating relapse of vivax malaria in IDP camps.

In addition to anti-malarials, vector intervention may have contributed in part to the low incidence rate in IDP camps. In this study, *An. minimus* was the predominant vector species [39]. Since *An. minimus* bites and rests both indoors and outdoors [42, 43], both ITNs and outdoor vector control interventions such as insecticide spray are important measures. In all study villages and camps, free ITNs were provided. However, ITNs in local villages were conventional ITN and were not re-treated routinely, whereas a high proportion of ITNs used in the IDP camps were LLINs that provided long-lasting protection. In addition, indoor and outdoor insecticide sprays were also provided free of charge, but this was performed more frequently in camps than in local villages. Insecticide spray was scheduled every few months in IDP camps. However, it was done only once a year during the summer in local villages, which was likely insufficient to suppress vector populations. Apart from insecticide spraying, management and treatment of mosquito breeding habitats, i.e., treating habitats with insecticides and/or draining the aquatic habitats, was also a routine work in the IDP camps. For example, more than 20 large water ponds (of which many were fish ponds) were observed in early 2013, and these ponds harboured a high density of mosquito larvae. Few months later in August, about half of these ponds were drained (Zhou, personal observation), and this has resulted in decreased adult density in IDP camp from 2013 to 2014. On the other hand, larval habitats have never been treated in local villages. The contrast in larval habitat management and indoor/outdoor preventive measures may explain the difference in malaria transmission between the IDP camp areas and local villages [10, 13, 14, 44–48]. The use of CDC light trap for vector surveillance may have biased to some species and underestimated the true vector density [49]. However, this is not a major problem because the same collection methods were used in all study sites for mosquito composition comparison. The higher malaria incidence rate in local residents may also be associated with the inaccessibility to health services in local residents. Although clinics in the IDP camps provide free treatment to local village residents, seeking treatment is not easy for them because of the distance and rough road conditions. Commute is especially difficult during rainy season from June to August, the onset of the high transmission season.

A decreased *P. falciparum* incidence rate in IDP camps but not in local villages, together with the increased trend in *P. vivax* over *P. falciparum* indicated effective treatment of falciparum malaria in IDP camps. Yet, despite these efforts, the camps experienced a *P. vivax* outbreak in 2013, a month after the peak in the villages. This is unlikely due to diagnostic errors given all infected cases diagnosed by microscopy and rapid diagnostic tests (RDT) were confirmed by PCR [38] method. Previous study in the same area showed that microscopic diagnoses had a sensitivity of about 75 % and specificity of 95 % for both vivax and falciparum infections compared to PCR. RDTs had a similar sensitivity to detect falciparum infections, but only 60 % of *P. vivax* infections [38]. The vivax epidemic might be due to the introduction of novel strains of *P. vivax* from the villages into the IDP camps whose residents have no prior immunity [40]. In contrast, the villages experienced a consistently high burden of malaria because no effective control measures or resources were available for this population. Lack of convenient access to health care facilities likely further accentuated this problem. Relief agencies and donors should expand healthcare services and malaria control measures to neglected communities beyond the resettled populations. Insufficient resource mobilization to areas that are difficult to access is a significant hurdle to disease control. The findings highlight the need of close monitoring and better healthcare in under-served and indigent populations residing in proximity to the IDP camps.

This study found that in local villages women of child-bearing age are at a higher risk of malaria compared to other women. The well-known adverse impacts of malaria during pregnancy warrant urgent attention to this vulnerable group [15–17, 50–53]. Globally, efforts to control and prevent malaria have primarily focused on *P. falciparum* in part because infections with this species pose a higher risk of mortality [54–56]. The emergence of a *P. vivax* outbreak in the IDP camps in 2013, despite sustained control measures and health care, highlights both the importance of *P. vivax* control in order to achieve malaria elimination [57, 58].

## Conclusion

Malaria in IDP camps can be significantly reduced with effective management. Despite sustained control measures and health care delivery, *P. vivax* outbreaks are unpredictable, which emphasizes both the difficulties and importance of *P. vivax* control in order to achieve malaria elimination. Expansion of malaria intervention strategies in IDP camps to local surrounding villages is critical to malaria control in the border area in particular and malaria elimination in China in general.

## Additional files

10.1186/s12936-016-1512-2 Number of household and total population in study villages/camps in August 2012.
10.1186/s12936-016-1512-2 Malaria incidence rates by parasite species.
10.1186/s12936-016-1512-2 Aid agencies, services they provided, and duration of their stay in different places.
10.1186/s12936-016-1512-2 Diseases prevention and control provided and medical services availabilities in different villages and camps.

## Abbreviations

IDP: internally displaced persons
CDC: Center for Diseases Prevention and Control
KIA: Kachin Independence Army
UNHCR: United Nations High Commissioner for Refugees
ICEMR: International Centers of Excellence for Malaria Research
NGO: non-governmental organization
PCS: passive case surveillance
ACS: active case surveillance
PCR: polymerase chain reaction
IRS: indoor residual spraying
ITN: insecticide-treated bed nets
ANOVA: analysis of variance
GEE: generalized estimating equations
OR: odds ratio
LLIN: long-lasting insecticide-treated nets
