# Supplementary material for: Impact of interventions on malaria in internally displaced persons along the China–Myanmar border: 2011–2014
Source: Malar J. 2016 Sep 15;15:471. doi: 10.1186/s12936-016-1512-2 (PMC5024476; doi:10.1186/s12936-016-1512-2)
Supplement: Supplementary file 1 — 10.1186/s12936-016-1512-2 Number of household and total population in study villages/camps in August 2012. [file 12936_2016_1512_MOESM1_ESM.docx]

Additional file 1. Number of household and total population in study villages/camps in August 2012.

| Study site | | Number of Households | Number of Individuals |
| --- | --- | --- | --- |
| No | Village/cam name |  |  |
| 1 | No. 3 Market camp | 348 | 1716 |
| 2 | Je Yang Hka camp | 1438 | 7332 |
| 3 | Hpun Lum Yang camp | 340 | 1634 |
| 4 | Shait Yang village | 25 | 106 |
| 5 | Mung Seng Yang village | 72 | 326 |
| 6 | Ja Htu Kawng village | 53 | 281 |
| 7 | Simsa Lawk village | 96 | 455 |
