# Supplementary material for: Impact of interventions on malaria in internally displaced persons along the China–Myanmar border: 2011–2014
Source: Malar J. 2016 Sep 15;15:471. doi: 10.1186/s12936-016-1512-2 (PMC5024476; doi:10.1186/s12936-016-1512-2)
Supplement: Supplementary file 2 — 10.1186/s12936-016-1512-2 Malaria incidence rates by parasite species. [file 12936_2016_1512_MOESM2_ESM.docx]

Additional file 2: Malaria incidence rates by parasite species

| Parasite species | Parameter | Category | IDP camp |  | Local village | Risk ratio |
| --- | --- | --- | --- | --- | --- | --- |
|  |  |  | Incidence rate |  | Incidence rate | Village/camp |
| *P. falciparum* | Overall |  | 3.08 |  | 21.39 | 6.94 *** |
|  | Gender | Male | 4.16 |  | 29.85 | 7.17 *** |
|  |  | Female | 2.30 |  | 12.95 | 5.62 *** |
|  | Age | 0~4 | 1.46 |  | 6.00 | 4.10 * |
|  |  | 5~14 | 2.97 |  | 18.22 | 6.14 *** |
|  |  | ≥15 | 3.90 |  | 26.26 | 6.73 *** |
|  | Female age | 15~45 | 2.41 |  | 18.15 | 7.54 *** |
|  |  | Other | 2.09 |  | 8.33 | 3.99 ** |
|  | Year | 2011 | 4.12 |  | 11.00 | 2.67 *** |
|  |  | 2012 | 3.58 |  | 18.06 | 5.05 *** |
|  |  | 2013 | 3.84 |  | 33.69 | 8.77 *** |
|  |  | 2014 | 1.21 |  | 20.67 | 17.14 *** |
| *P. vivax* | Overall |  | 35.10 |  | 70.11 | 2.00 *** |
|  | Gender | Male | 39.26 |  | 93.70 | 2.39 *** |
|  |  | Female | 34.50 |  | 46.88 | 1.36 ** |
|  | Age | 0~4 | 32.55 |  | 82.00 | 2.52 *** |
|  |  | 5~14 | 65.60 |  | 58.31 | 0.89 |
|  |  | ≥15 | 21.54 |  | 75.54 | 3.51 *** |
|  | Female age | 15~45 | 25.07 |  | 47.38 | 1.89 ** |
|  |  | Other | 38.03 |  | 43.94 | 1.32 |
|  | Year | 2011 | 8.61 |  | 16.50 | 1.92 *** |
|  |  | 2012 | 11.51 |  | 53.31 | 4.63 *** |
|  |  | 2013 | 83.97 |  | 106.43 | 1.27 |
|  |  | 2014 | 29.51 |  | 96.47 | 3.27 *** |
