# Supplementary material for: Impact of interventions on malaria in internally displaced persons along the China–Myanmar border: 2011–2014
Source: Malar J. 2016 Sep 15;15:471. doi: 10.1186/s12936-016-1512-2 (PMC5024476; doi:10.1186/s12936-016-1512-2)
Supplement: Supplementary file 3 — 10.1186/s12936-016-1512-2 Aid agencies, services they provided, and duration of their stay in different places. [file 12936_2016_1512_MOESM3_ESM.docx]

Additional file 3. Aid agencies, services they provided, and duration of their stay in different places.

| Village | Name and type of agency | Start year | Duration of stay † | Service(s) provided | | | | | | |
| --- | --- | --- | --- | --- | --- | --- | --- | --- | --- | --- |
|  |  |  |  | ITN | Medical | Water, sanitation | House construction | Food supply | Daily necessities | Other § |
| Je Yang camp | | | |  |  |  |  |  |  |  |
|  | UNCHR | 2013 | < 1 |  |  | √ |  |  |  |  |
|  | IRRC local team ‡ | 2011 | 12 |  |  |  | √ | √ |  |  |
|  | KRDC local government ‡ | 2011 | 12 |  |  |  |  | √ | √ |  |
|  | H.U. Kachin Project ‡ | 2011 | 12 | √ | √ | √ |  |  | √ |  |
|  | KMSS | 2011 | < 1 |  |  |  | √ |  | √ |  |
|  | HPA | 2011 | < 1 |  | Supplies |  |  |  |  | √ |
|  | Myanmar Zin Lur | 2012 | < 1 |  |  | √ |  |  | √ |  |
|  | Dehong Church | 2013 | < 1 |  |  |  |  |  |  | √ |
|  | Singapore church | 2013 | < 1 |  |  |  |  |  |  | √ |
|  | Shanghai Baptist Church | 2013 | < 1 | √ 1000 |  |  |  |  |  |  |
|  | U.S.A. | 2012 | < 1 |  | √ |  |  |  |  |  |
|  | Kachin Diploment Group | 2013 | < 1 |  |  |  |  |  | √ |  |
| Hpum Lum Yang camp | |  |  |  |  |  |  |  |  |  |
|  | UNCHR | 2013 | < 1 | √ | Supplies |  |  |  | √ |  |
|  | UNICEF | 2011 | < 1 | √ |  | √ |  |  | √ |  |
|  | KRDC local government ‡ | 2012 | 12 |  |  | √ | √ | √ |  | √ |
|  | H.U. Kachin Project ‡ | 2011 | 12 | √ | √ | √ |  | √ | √ |  |
|  | World Food Programme | 2013 | < 1 |  |  |  |  | √ |  |  |
|  | Shalom, local organization | 2013 | < 1 |  |  |  |  |  |  | √ |
|  | KIC | 2012 | < 1 |  |  | √ |  |  |  | √ |
|  | Expiration Myanmar | 2013 | < 1 |  |  |  |  |  | √ | √ |
|  | Yangon Group | 2013 | < 1 |  |  |  |  | √ |  | √ |
|  | Fujian-Zhejiang aid | 2013 | < 1 |  |  |  |  |  | √ | √ |
|  | Bamer Riger aid | 2013 | < 1 |  |  |  |  |  |  | √ |
| Ja Htu Kawng | | |  |  |  |  |  |  |  |  |
|  | UNCHR | 2013 | < 1 | √ |  |  |  | √ | √ |  |
|  | KBC | 2013 | < 1 |  |  |  |  |  | √ |  |
|  | Myanmar aid | 2013 | < 1 |  |  |  |  |  | √ | √ |
| Mung Seng Yang | |  |  |  |  |  |  |  |  |  |
|  | UNCHR | 2013 | < 1 |  |  |  |  |  | √ |  |
|  | KMSS | 2013 | < 1 |  |  |  |  |  | √ | √ |

† Number of months per year.

‡ These agencies stayed permanently.

§ School supplies and financial support.
