# Supplementary material for: Impact of interventions on malaria in internally displaced persons along the China–Myanmar border: 2011–2014
Source: Malar J. 2016 Sep 15;15:471. doi: 10.1186/s12936-016-1512-2 (PMC5024476; doi:10.1186/s12936-016-1512-2)
Supplement: Supplementary file 4 — 10.1186/s12936-016-1512-2 Diseases prevention and control provided and medical services availabilities in different villages and camps. [file 12936_2016_1512_MOESM4_ESM.docx]

Additional file 4. Diseases prevention and control provided and medical services availabilities in different villages and camps.

| Village/Camp name | |  | Je Yang camp | Hpum Lum Yang camp | Ja Htu Kawng † | Mung Seng Yang† |
| --- | --- | --- | --- | --- | --- | --- |
| Diseases control activities | | |  |  |  |  |
|  | List of activities |  | Insecticide spray, ITN, drainage clean up | Insecticide spray, ITN | Insecticide spray, ITN | Insecticide spray |
|  | Places sprayed | Indoor wall/ceiling | Yes | Yes | Yes | Yes |
|  |  | Outdoor ground | Yes | Yes | Yes | Yes |
|  |  | Outdoor waterbody | Yes | Yes | No | Yes |
|  |  | Outdoor other place | Yes | Yes | No | Yes |
|  | Frequency of spray | | Any time if needed | once every 3 months | once in summer | once in summer |
| Free bed net | |  | To everybody | To everybody | To everybody | No |
| Clinic/hospital in village/camp | | | Yes | Yes | No | No |
|  | Number of doctors | | 2 | 0 | N/A † | N/A† |
|  | Number of nurses | | 15 | 5 | N/A | N/A |
|  | Microscope |  | Yes | Yes | N/A | N/A |
|  | Free diagnosis |  | Yes | Yes | Yes | No |
|  | Free medicine |  | Yes | Yes | Yes | No |
|  | Free vaccination | | Yes | Yes | Yes | No |
|  | Free malaria treatment | | Yes | Yes | Yes | No |
|  | Shortage of supplies | | Yes | Yes | N/A | N/A |

† Medical services occasionally available at military hospitals about 1.5 km from the village.

‡ N/A – not applicable.
